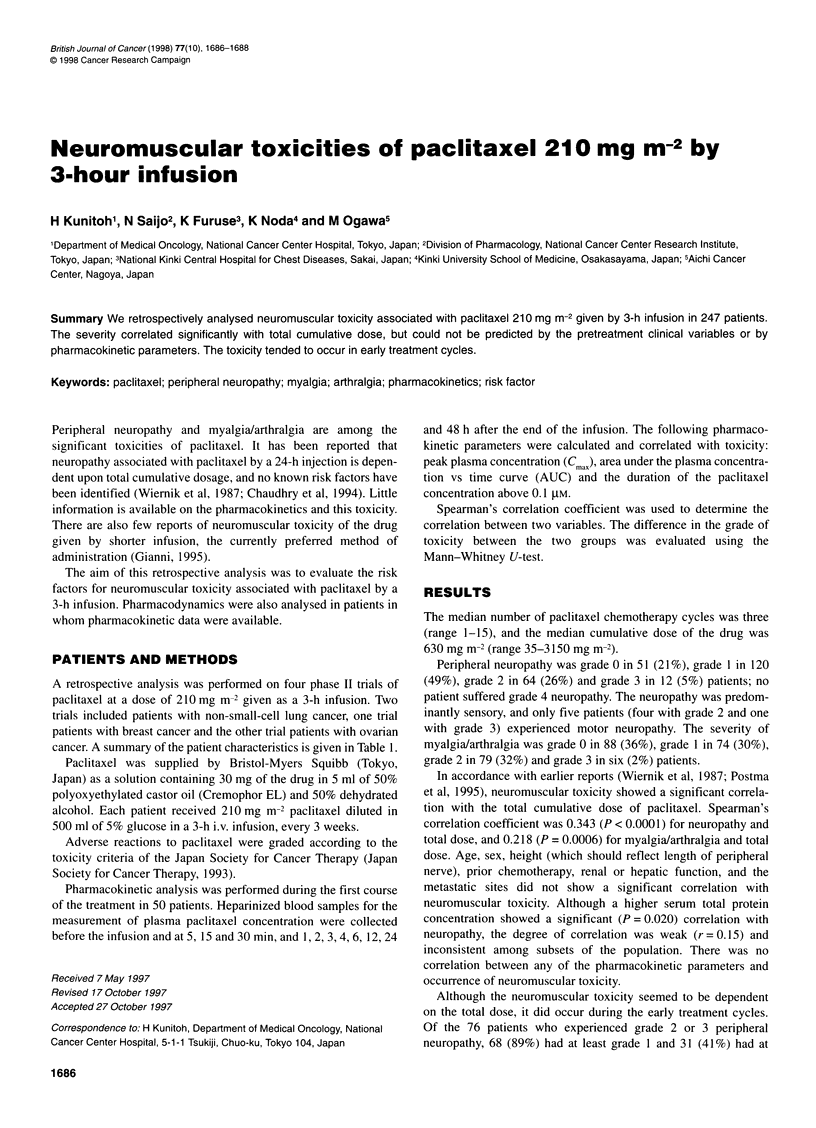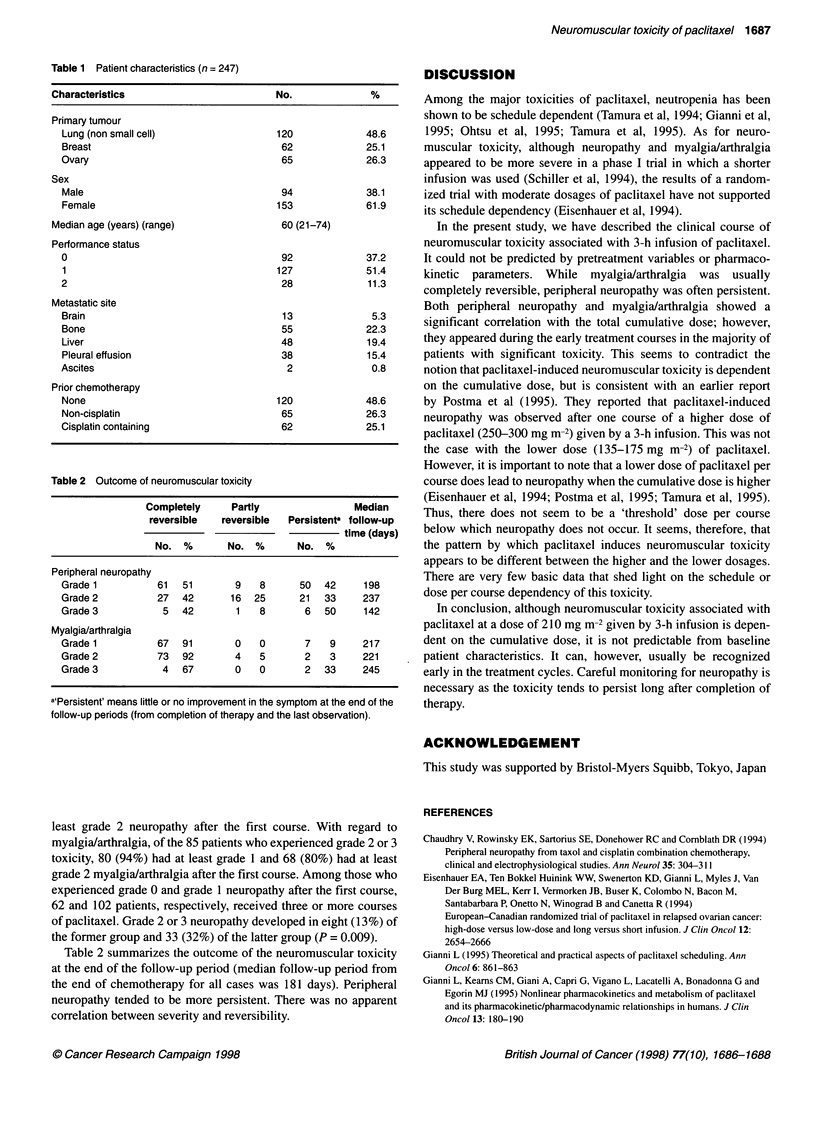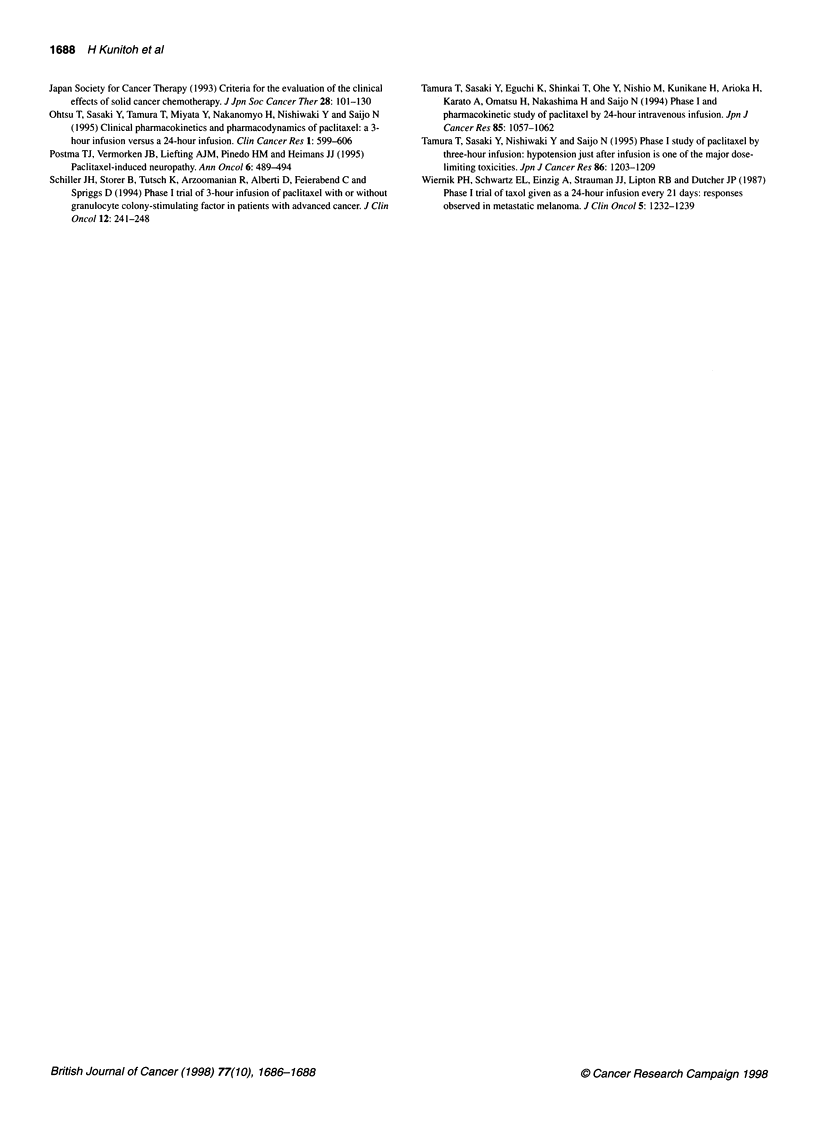# Neuromuscular toxicities of paclitaxel 210 mg m(-2) by 3-hour infusion.

**DOI:** 10.1038/bjc.1998.278

**Published:** 1998-05

**Authors:** H. Kunitoh, N. Saijo, K. Furuse, K. Noda, M. Ogawa

**Affiliations:** Department of Medical Oncology, National Cancer Center Hospital, Tokyo, Japan.

## Abstract

We retrospectively analysed neuromuscular toxicity associated with paclitaxel 210 mg m(-2) given by 3-h infusion in 247 patients. The severity correlated significantly with total cumulative dose, but could not be predicted by the pretreatment clinical variables or by pharmacokinetic parameters. The toxicity tended to occur in early treatment cycles.


British Journal of Cancer (1998) 77(10), 1686–1688
© 1998 Cancer Research Campaign

### Neuromuscular toxicities of paclitaxel 210 mg m$^{-2}$ by 3-hour infusion

**H Kunitoh[1], N Saijo[2], K Furuse[3], K Noda[4] and M Ogawa[5]**

[1]Department of Medical Oncology, National Cancer Center Hospital, Tokyo, Japan; [2]Division of Pharmacology, National Cancer Center Research Institute, Tokyo, Japan; [3]National Kinki Central Hospital for Chest Diseases, Sakai, Japan; [4]Kinki University School of Medicine, Osakasayama, Japan; [5]Aichi Cancer Center, Nagoya, Japan

**Summary** We retrospectively analysed neuromuscular toxicity associated with paclitaxel 210 mg m$^{-2}$ given by 3-h infusion in 247 patients. The severity correlated significantly with total cumulative dose, but could not be predicted by the pretreatment clinical variables or by pharmacokinetic parameters. The toxicity tended to occur in early treatment cycles.

**Keywords:** paclitaxel; peripheral neuropathy; myalgia; arthralgia; pharmacokinetics; risk factor

Peripheral neuropathy and myalgia/arthralgia are among the significant toxicities of paclitaxel. It has been reported that neuropathy associated with paclitaxel by a 24-h injection is dependent upon total cumulative dosage, and no known risk factors have been identified (Wiernik et al, 1987; Chaudhry et al, 1994). Little information is available on the pharmacokinetics and this toxicity. There are also few reports of neuromuscular toxicity of the drug given by shorter infusion, the currently preferred method of administration (Gianni, 1995).

The aim of this retrospective analysis was to evaluate the risk factors for neuromuscular toxicity associated with paclitaxel by a 3-h infusion. Pharmacodynamics were also analysed in patients in whom pharmacokinetic data were available.

### PATIENTS AND METHODS

A retrospective analysis was performed on four phase II trials of paclitaxel at a dose of 210 mg m$^{-2}$ given as a 3-h infusion. Two trials included patients with non-small-cell lung cancer, one trial patients with breast cancer and the other trial patients with ovarian cancer. A summary of the patient characteristics is given in Table 1.

Paclitaxel was supplied by Bristol-Myers Squibb (Tokyo, Japan) as a solution containing 30 mg of the drug in 5 ml of 50% polyoxyethylated castor oil (Cremophor EL) and 50% dehydrated alcohol. Each patient received 210 mg m$^{-2}$ paclitaxel diluted in 500 ml of 5% glucose in a 3-h i.v. infusion, every 3 weeks.

Adverse reactions to paclitaxel were graded according to the toxicity criteria of the Japan Society for Cancer Therapy (Japan Society for Cancer Therapy, 1993).

Pharmacokinetic analysis was performed during the first course of the treatment in 50 patients. Heparinized blood samples for the measurement of plasma paclitaxel concentration were collected before the infusion and at 5, 15 and 30 min, and 1, 2, 3, 4, 6, 12, 24 and 48 h after the end of the infusion. The following pharmacokinetic parameters were calculated and correlated with toxicity: peak plasma concentration ($C_{max}$), area under the plasma concentration vs time curve (AUC) and the duration of the paclitaxel concentration above 0.1 μM.

Spearman's correlation coefficient was used to determine the correlation between two variables. The difference in the grade of toxicity between the two groups was evaluated using the Mann–Whitney $U$-test.

*Received 7 May 1997*
*Revised 17 October 1997*
*Accepted 27 October 1997*

*Correspondence to:* H Kunitoh, Department of Medical Oncology, National Cancer Center Hospital, 5-1-1 Tsukiji, Chuo-ku, Tokyo 104, Japan

### RESULTS

The median number of paclitaxel chemotherapy cycles was three (range 1–15), and the median cumulative dose of the drug was 630 mg m$^{-2}$ (range 35–3150 mg m$^{-2}$).

Peripheral neuropathy was grade 0 in 51 (21%), grade 1 in 120 (49%), grade 2 in 64 (26%) and grade 3 in 12 (5%) patients; no patient suffered grade 4 neuropathy. The neuropathy was predominantly sensory, and only five patients (four with grade 2 and one with grade 3) experienced motor neuropathy. The severity of myalgia/arthralgia was grade 0 in 88 (36%), grade 1 in 74 (30%), grade 2 in 79 (32%) and grade 3 in six (2%) patients.

In accordance with earlier reports (Wiernik et al, 1987; Postma et al, 1995), neuromuscular toxicity showed a significant correlation with the total cumulative dose of paclitaxel. Spearman's correlation coefficient was 0.343 ($P < 0.0001$) for neuropathy and total dose, and 0.218 ($P = 0.0006$) for myalgia/arthralgia and total dose. Age, sex, height (which should reflect length of peripheral nerve), prior chemotherapy, renal or hepatic function, and the metastatic sites did not show a significant correlation with neuromuscular toxicity. Although a higher serum total protein concentration showed a significant ($P = 0.020$) correlation with neuropathy, the degree of correlation was weak ($r = 0.15$) and inconsistent among subsets of the population. There was no correlation between any of the pharmacokinetic parameters and occurrence of neuromuscular toxicity.

Although the neuromuscular toxicity seemed to be dependent on the total dose, it did occur during the early treatment cycles. Of the 76 patients who experienced grade 2 or 3 peripheral neuropathy, 68 (89%) had at least grade 1 and 31 (41%) had at

**Table 1** Patient characteristics ($n$ = 247)

| Characteristics | No. | % |
|---|---|---|
| Primary tumour | | |
| Lung (non small cell) | 120 | 48.6 |
| Breast | 62 | 25.1 |
| Ovary | 65 | 26.3 |
| Sex | | |
| Male | 94 | 38.1 |
| Female | 153 | 61.9 |
| Median age (years) (range) | 60 (21–74) | |
| Performance status | | |
| 0 | 92 | 37.2 |
| 1 | 127 | 51.4 |
| 2 | 28 | 11.3 |
| Metastatic site | | |
| Brain | 13 | 5.3 |
| Bone | 55 | 22.3 |
| Liver | 48 | 19.4 |
| Pleural effusion | 38 | 15.4 |
| Ascites | 2 | 0.8 |
| Prior chemotherapy | | |
| None | 120 | 48.6 |
| Non-cisplatin | 65 | 26.3 |
| Cisplatin containing | 62 | 25.1 |

**Table 2** Outcome of neuromuscular toxicity

| | Completely reversible | | Partly reversible | | Persistent[a] | | Median follow-up time (days) |
|---|---|---|---|---|---|---|---|
| | No. | % | No. | % | No. | % | |
| Peripheral neuropathy | | | | | | | |
| Grade 1 | 61 | 51 | 9 | 8 | 50 | 42 | 198 |
| Grade 2 | 27 | 42 | 16 | 25 | 21 | 33 | 237 |
| Grade 3 | 5 | 42 | 1 | 8 | 6 | 50 | 142 |
| Myalgia/arthralgia | | | | | | | |
| Grade 1 | 67 | 91 | 0 | 0 | 7 | 9 | 217 |
| Grade 2 | 73 | 92 | 4 | 5 | 2 | 3 | 221 |
| Grade 3 | 4 | 67 | 0 | 0 | 2 | 33 | 245 |

[a]'Persistent' means little or no improvement in the symptom at the end of the follow-up periods (from completion of therapy and the last observation).

least grade 2 neuropathy after the first course. With regard to myalgia/arthralgia, of the 85 patients who experienced grade 2 or 3 toxicity, 80 (94%) had at least grade 1 and 68 (80%) had at least grade 2 myalgia/arthralgia after the first course. Among those who experienced grade 0 and grade 1 neuropathy after the first course, 62 and 102 patients, respectively, received three or more courses of paclitaxel. Grade 2 or 3 neuropathy developed in eight (13%) of the former group and 33 (32%) of the latter group ($P$ = 0.009).

Table 2 summarizes the outcome of the neuromuscular toxicity at the end of the follow-up period (median follow-up period from the end of chemotherapy for all cases was 181 days). Peripheral neuropathy tended to be more persistent. There was no apparent correlation between severity and reversibility.

### DISCUSSION

Among the major toxicities of paclitaxel, neutropenia has been shown to be schedule dependent (Tamura et al, 1994; Gianni et al, 1995; Ohtsu et al, 1995; Tamura et al, 1995). As for neuromuscular toxicity, although neuropathy and myalgia/arthralgia appeared to be more severe in a phase I trial in which a shorter infusion was used (Schiller et al, 1994), the results of a randomized trial with moderate dosages of paclitaxel have not supported its schedule dependency (Eisenhauer et al, 1994).

In the present study, we have described the clinical course of neuromuscular toxicity associated with 3-h infusion of paclitaxel. It could not be predicted by pretreatment variables or pharmacokinetic parameters. While myalgia/arthralgia was usually completely reversible, peripheral neuropathy was often persistent. Both peripheral neuropathy and myalgia/arthralgia showed a significant correlation with the total cumulative dose; however, they appeared during the early treatment courses in the majority of patients with significant toxicity. This seems to contradict the notion that paclitaxel-induced neuromuscular toxicity is dependent on the cumulative dose, but is consistent with an earlier report by Postma et al (1995). They reported that paclitaxel-induced neuropathy was observed after one course of a higher dose of paclitaxel (250–300 mg $m^{-2}$) given by a 3-h infusion. This was not the case with the lower dose (135–175 mg $m^{-2}$) of paclitaxel. However, it is important to note that a lower dose of paclitaxel per course does lead to neuropathy when the cumulative dose is higher (Eisenhauer et al, 1994; Postma et al, 1995; Tamura et al, 1995). Thus, there does not seem to be a 'threshold' dose per course below which neuropathy does not occur. It seems, therefore, that the pattern by which paclitaxel induces neuromuscular toxicity appears to be different between the higher and the lower dosages. There are very few basic data that shed light on the schedule or dose per course dependency of this toxicity.

In conclusion, although neuromuscular toxicity associated with paclitaxel at a dose of 210 mg $m^{-2}$ given by 3-h infusion is dependent on the cumulative dose, it is not predictable from baseline patient characteristics. It can, however, usually be recognized early in the treatment cycles. Careful monitoring for neuropathy is necessary as the toxicity tends to persist long after completion of therapy.

### ACKNOWLEDGEMENT

This study was supported by Bristol-Myers Squibb, Tokyo, Japan

### REFERENCES

Chaudhry V, Rowinsky EK, Sartorius SE, Donehower RC and Cornblath DR (1994) Peripheral neuropathy from taxol and cisplatin combination chemotherapy, clinical and electrophysiological studies. *Ann Neurol* **35**: 304–311

Eisenhauer EA, Ten Bokkel Huinink WW, Swenerton KD, Gianni L, Myles J, Van Der Burg MEL, Kerr I, Vermorken JB, Buser K, Colombo N, Bacon M, Santabarbara P, Onetto N, Winograd B and Canetta R (1994) European–Canadian randomized trial of paclitaxel in relapsed ovarian cancer: high-dose versus low-dose and long versus short infusion. *J Clin Oncol* **12**: 2654–2666

Gianni L (1995) Theoretical and practical aspects of paclitaxel scheduling. *Ann Oncol* **6**: 861–863

Gianni L, Kearns CM, Giani A, Capri G, Vigano L, Lacatelli A, Bonadonna G and Egorin MJ (1995) Nonlinear pharmacokinetics and metabolism of paclitaxel and its pharmacokinetic/pharmacodynamic relationships in humans. *J Clin Oncol* **13**: 180–190

© Cancer Research Campaign 1998 

Japan Society for Cancer Therapy (1993) Criteria for the evaluation of the clinical effects of solid cancer chemotherapy. *J Jpn Soc Cancer Ther* **28**: 101–130

Ohtsu T, Sasaki Y, Tamura T, Miyata Y, Nakanomyo H, Nishiwaki Y and Saijo N (1995) Clinical pharmacokinetics and pharmacodynamics of paclitaxel: a 3-hour infusion versus a 24-hour infusion. *Clin Cancer Res* **1**: 599–606

Postma TJ, Vermorken JB, Liefting AJM, Pinedo HM and Heimans JJ (1995) Paclitaxel-induced neuropathy. *Ann Oncol* **6**: 489–494

Schiller JH, Storer B, Tutsch K, Arzoomanian R, Alberti D, Feierabend C and Spriggs D (1994) Phase I trial of 3-hour infusion of paclitaxel with or without granulocyte colony-stimulating factor in patients with advanced cancer. *J Clin Oncol* **12**: 241–248

Tamura T, Sasaki Y, Eguchi K, Shinkai T, Ohe Y, Nishio M, Kunikane H, Arioka H, Karato A, Omatsu H, Nakashima H and Saijo N (1994) Phase I and pharmacokinetic study of paclitaxel by 24-hour intravenous infusion. *Jpn J Cancer Res* **85**: 1057–1062

Tamura T, Sasaki Y, Nishiwaki Y and Saijo N (1995) Phase I study of paclitaxel by three-hour infusion: hypotension just after infusion is one of the major dose-limiting toxicities. *Jpn J Cancer Res* **86**: 1203–1209

Wiernik PH, Schwartz EL, Einzig A, Strauman JJ, Lipton RB and Dutcher JP (1987) Phase I trial of taxol given as a 24-hour infusion every 21 days: responses observed in metastatic melanoma. *J Clin Oncol* **5**: 1232–1239

 *© Cancer Research Campaign 1998*